# Structure, Oxygen Content and Electric Properties of Titanium Nitride Electrodes in TiN_x_/La:HfO_2_/TiN_x_ Stacks Grown by PEALD on SiO_2_/Si

**DOI:** 10.3390/nano12203608

**Published:** 2022-10-14

**Authors:** Elena I. Suvorova, Oleg V. Uvarov, Kirill V. Chizh, Alexey A. Klimenko, Philippe A. Buffat

**Affiliations:** 1 A.V. Shubnikov Institute of Crystallography, Federal Scientific Research Centre “Crystallography and Photonics” of the Russian Academy of Sciences, Leninsky pr. 59, Moscow 119333, Russia; 2 Prokhorov General Physics Institute of the Russian Academy of Sciences, Vavilov str. 38, Moscow 119991, Russia; 3 Institute of Nanotechnology of Microelectronics of the Russian Academy of Sciences, Leninsky pr. 32A, Moscow 119991, Russia; 4 Ecole Polytechnique Fédérale de Lausanne, Centre Interdisciplinaire de Microscopie Electronique, CH-1015 Lausanne, Switzerland

**Keywords:** titanium nitride, titanium oxynitride, MIM stack, transmission electron microscopy, energy dispersive X-ray spectrometry, electron energy loss spectroscopy, band gap, resistivity

## Abstract

This work reports experimental results of the quantitative determination of oxygen and band gap measurement in the TiN_x_ electrodes in planar TiN_x_ top/La:HfO_2_/TiN_x_ bottom MIM stacks obtained by plasma enhanced atomic layer deposition on SiO_2_. Methodological aspects of extracting structural and chemical information from (scanning) transmission electron microscopy imaging (bright field and high angular annular dark field), energy dispersive X-ray spectrometry and electron energy loss spectroscopy are thoroughly considered. The study shows that the oxygen concentration is higher in the TiN_x_O_y_ bottom electrode (about 14.2 ± 0.1 at. %) compared to the TiN_x_O_y_ top electrode (about 11.4 ± 0.5 at. %). The following average stoichiometric formulas are TiN_0_._52_O_0_._20_ top and TiN_0_._54_O_0_._26_ bottom for top and bottom electrodes, respectively. The amount of oxygen incorporated into TiN_x_ during PEALD because of oxygen impurities in the plasma is minor compared to that because of diffusion from SiO_2_ and HfO_2_. This asymmetry, together with results on a sample grown on a Si substrate, shows that incorporating oxygen impurity from the plasma itself is a minor part compared to diffusion from the SiO_2_ substrate and HfO_2_ dielectric during the PEALD growth. We observe the presence of TiO_2_ at the interface between the Hf oxide layer and the Ti nitride electrodes as well as at the SiO_2_ interface. EELS analysis led to a band gap ranging from 2.2 to 2.5 eV for the bottom TiN_x_O_y_ and 1.7–2.2 eV for the top TiN_x_O_y_, which is in fair agreement with results obtained on the top TiN_x_ electrode (1.6 ± 01 eV) using optical absorption spectra. Measurement of sheet resistance, resistivity and temperature coefficient of resistance by a four-point probe on the top TiN_x_O_y_ electrode from 20 to 100 °C corresponds to the typical values for semiconductors.

## 1. Introduction

Improving the stability and the strength of metal-insulator-metal (MIM) capacitors in integrated circuits without increasing their size still remains a significant challenge for reliability, performance and cost. Among various metal-oxide-based materials for MIM capacitors for ReRAM (random access resistive memory) or FeRAM (ferroelectric RAM), HfO_2_/ZrO_2_ has drawn a large amount attention and has been studied extensively. The reason is that titanium nitride hafnium oxide-based materials/titanium nitride capacitors can participate in minimizing the size of devices and be able to create three-dimensional (3D) capacitors in the opposite, for instance, perovskite-based FeRAM capacitors [1,2,3]. Titanium nitride layers obtained by the atomic layer deposition (ALD) method possess high thermodynamic stability, high mechanical hardness and low electrical resistance. They are low-cost electrode materials that, in addition, have a high degree of conformal coating of steps on devices with non-planar geometry.

However, among the known problems of such MIM structures, the wake-up effect and insufficient endurance are critical, and the structure and composition of materials (dielectrics and electrodes) have a strong influence on the device properties [4,5]. Despite its advantages, titanium nitride exhibits properties such as non-stoichiometry and sensitivity to oxidation, which makes it challenging to use.

Based on titanium nitride’s electronic structure and chemical bonding characteristics, TiN is classified among the interstitial nitrides [6]. It is able to host very different amounts of nitrogen, and it is usually expressed as TiN_x_ or TiN instead of TiN_x_. It keeps the TiN face-centered cubic (FCC) close-packed structure (sp.gr. Fm-3m, a ≈ 4.243 Å) with only a small change in lattice parameters in the range TiN_0_._4_ to TiN_1_._1_ [4,7], but a significant change in its physical properties. The electrical properties are strongly dependent on the non-stoichiometry and defective structure of titanium nitride. In particular, the presence of vacancies instead of nitrogen reduces the number of conduction electrons and increases the electrical resistivity [8].

TiN layers can take up substantial amounts of oxygen, either from the environment or the dielectric layer during processing [9,10,11]. Therefore, the formation of defective interfacial phases between metal oxides and electrodes soon became a serious problem. Jeon [12] reports that the formation of TiON at the TiN/oxide dielectric interfaces is responsible for an increase in the leakage current and for a capacitance degradation in MIM. The degradation can be caused by the formation of defects in the dielectric layer [13] due to the scavenging of oxygen from HfO_2_ [14,15]. Therefore, special attention is paid to the electrode/dielectric interface structure, quality, roughness and chemical composition, which altogether affect the electrical behavior of MIM capacitors [16,17,18,19,20,21]. Interfaces of TiN_x_ electrodes with HfO_2_ (or Hf_0_._5_Zr_0_._5_O_2_) layers are being intensively investigated to assess the possible presence of TiN_x_O_y_ and TiO_2_ phases [13,21,22].

At elevated temperatures of 350–450 °C, N-deficient TiNx transforms to (non)stoichiometric titanium oxynitride (TiN_x_O_y_) [23,24]. TiN_x_O_y_ films change their electrical and optical properties with oxygen and nitrogen contents ranging between 0 ≤ x ≤ 1.0 and 0 ≤ y ≤ 2.0 and undergo a transition from metallic to semiconducting behavior [1,25]. In this latter case, the dielectric permittivity of TiO_x_N_y_ (≈5) reduces the capacitance of the MIM structure [26]. Despite a significant number of publications devoted to the dependence of the properties of titanium nitride on its composition, very often the precise compositions of the material itself remain unknown and are replaced by “more” or “less”. Our work focuses on the relationship between the precise multilayer structure and composition and properties of titanium nitride in MIM capacitors, in particular, the electrode body and interfaces.

The precursors and added gas during plasma-enhanced atomic layer deposition (PEALD), as well as the remaining atmosphere and even the nature of the reaction chamber wall, are most often considered the main sources of oxygen inside the titanium nitride layer. We will show that oxygen diffusion from the surface (natural aging or post-growth annealing) would not be compatible with the concentration profiles from the surface to the substrate. Moreover, the comparison between two samples, one grown on SiO_2_ and the other on Si with a native oxide of only 1.5 nm deep, shows that the oxygen content in the adjacent electrode depends on the oxygen availability in the substrate. This provides evidence that at least a large part of the oxygen stems from the neighboring oxide layers, and diffusion should be considered as a pathway for the formation of titanium oxynitride. Diffusion inside the TiN_x_ layer enhanced by intergranular boundaries in its columnar structure has been reported [27,28,29] but does not explain oxygen availability. Therefore, the actual mechanism of this diffusion still needs more investigation.

Most often, microelectronics researchers and technologists base their knowledge of the structures on information gathered indirectly from the free surface of the samples with a deconvolution process for depth profiling or using sputtering for in-depth investigation, for example, X-ray photoelectron spectroscopy (XPS), grazing angle X-ray diffraction (GIXRD), secondary ion mass spectrometry (SIMS), Rutherford backscattering spectroscopy (RBS and HR-RBS). Alternatively, electron microscopy on sample cross-sections provides direct access to in-depth views and profiles with a higher 3-D spatial resolution, that confirm, refine and complement the information available from the surface approach. In addition, it reveals the interface roughness and its amplitude and brings information necessary for analytical interpretation.

Ignoring the presence of roughness at an interface between two phases A and B would lead to the conclusion of a new phase AB. Taking into account the roughness, a crude approach does not allow distinguishing a mixture of A and B phases from a new AB phase or even from a mixture of the three. This ambiguity can be solved with analytical electron microscopy by combining high-angle dark field (HAADF) images, energy dispersive X-ray spectrometry (EDXS) and energy loss spectroscopy (EELS). However, the overall composition of the present sample, Si-O-Ti-N-Hf-Zr-La with a Pt-C cap, implies several characteristic X-ray line overlaps and a wide range of energy edges in EELS that require paying careful attention to the methodology. The aim of this work, beyond an overall quantitative analysis of the distribution of all present elements, was the confirmation of the presence of oxygen in the TiN_x_ electrodes and at their interfaces with hafnium and silicon oxide, its local concentration and in which phase (TiN_x_ plus TiO_2_ or single oxynitride TiN_x_O_y_). Due to the interfacial roughness, this last point was only solved by EELS analysis. Low-loss energy spectroscopy was also performed to derive the band gap of TiN_x_ across the whole thickness of the electrodes in the MIM structures.

This paper reports the results obtained for the two different kinds of MIM structures (which are part of a development project for ferroelectric RAM devices) that have shown the best electrophysical properties: (i) the TiN_x_/La:HfO_2_/TiN_x_ structure on SiO_2_ annealed 30 s at 650 °C, which has a maximum remnant polarization 2Pr ≈ 34 C/cm^2^, (ii) the Pt/Hf_0_._5_Zr_0_._5_O_2_/TiN_x_ structure on (100) Si annealed 30 s at 550 °C showed a 2Pr ≈ 32 C/cm^2^. The present experimental results should provide essential elements for the improvement of existing theoretical models of oxygen transport in titanium nitride layers in MIM capacitors.

## 2. Materials and Methods

Planar 20 nm TiN_x_ (top electrode)/10 nm La:HfO_2_/20 nm TiN_x_ (bottom electrode) MIM capacitors were grown on 150 mm diameter Si wafers coated with a 1 µm thick SiO_2_ layer created by wet oxidation method. The top and bottom TiNx layers were deposited from TiCl_4_ and NH_3_ precursors in a pulsed plasma (plasma enhanced atomic layer deposition, PEALD) on the SiO_2_ substrate and the La-HfO_2_ layer respectively at 330 °C. The La-doped HfO_2_ layers were obtained by PEALD from Hf(NC_2_H_5_CH_3_)_4_, La(i-C_3_H_7_C_5_H_4_)_3_ precursors in an O_2_ plasma at substrate temperature 235 °C. Lastly a rapid thermal processing was applied under nitrogen at 550, 650 or 750 °C for 30 s, all the details of the synthesis can be found in [30].

To track a possible contribution from the SiO_2_ substrate to the oxygen content in the bottom TiN_x_ layer, a 4 nm Pt/10 nm Hf_0_._5_Zr_0_._5_O_2_/20 nm TiN_x_ structure on a Si substrate with the unavoidable thin native oxide SiO_x_ layer (about 1.5 nm thick) [31] was also studied. The same growth procedure as above was applied with exception of a replacement of the La(i-C_3_H_7_C_5_H_4_)_3_ precursor by Zr(NC_2_H_5_CH_3_)_4_ for the dielectric, the addition of a Pt cap and a post-treatment in 450–600 °C under N_2_ for 30 s.

TEM (transmission electron microscopy) thin cross-sections were prepared either with focused ion beam (FEI Scios FIB) or conventional Ar ion milling (Gatan PIPS, Pleasanton, CA, USA) at room temperature after preliminary mechanical polishing. TEM and STEM (scanning TEM) images, EDXS microanalysis and selected area electron diffraction (SAED) patterns were obtained at 200 kV accelerating voltage on an analytical field emission transmission electron microscope (FEI Tecnai Osiris, Hillsboro, OR, USA) fitted with a 4 SDD detectors Super X ChemiSTEM EDXS system (FEI) running under ESPRIT software (Bruker).

Crystallographic phase analysis was performed using the selected area electron diffraction (SAED) patterns and Fourier transform diffractograms (FFT) of HRTEM (high-resolution TEM) images, which were processed with the DigitalMicrograph suite GMS 2.31 (Gatan Inc, Pleasanton, CA, USA). SAED patterns and FFT were interpreted with the JEMS software [32]. Accurate element quantification requires an estimate of sample thickness for X-Ray absorption correction. Assuming this thickness is constant over the whole area of interest, it was estimated by matching JEMS simulations to HRTEM images when available [33] or trial and error until the Cliff–Lorimer quantification was matching the HfO_2_ or HfO_0_._5_Zr_0_._5_O_2_ stoichiometry considering that these layers did not contain large number of foreign elements.

EELS spectra were obtained on a Libra 200 FE HR 200 kV microscope (Zeiss, Oberkochen, Germany) with an in-column EELS filter at 200 kV accelerating voltage (energy resolution was 0.7 eV, energy dispersion of 0.06 eV/channel, illumination convergence semi-angle α = 15 mrad and collection semi-angle β = 20 mrad). Several sets of spectra were collected in STEM mode either across the multilayer structure from the SiO_2_ layer up to the TiN_x_ top free surface or along the layer medians of the TiN_x_ bottom, La:HfO_2_ and TiN_x_ top layers, respectively. The core loss EELS spectra were used to determine the titanium, oxygen and nitrogen concentrations in TiN_x_ layers after subtraction of a power law model of background. The plural scattering and the contribution of the zero-loss peak tail of the energy low-loss spectra were removed by deconvolution with a Fourier-log approach in DigitalMicrograph 2.31 to obtain band gap evaluation.

EDXS and EELS provide complementary information on the spatial, crystallographic and chemical structure, with additional access to electronic properties for the latter.

EDXS, in comparison with EELS, has the major advantage of a global view of all present chemical elements in a single spectrum, from large areas down to the nanometer scale and even below. At first glance, the built-in software gives access to instant and effortless quantitative elemental analysis. In comparison, each EELS spectrum covers only a small part of the energy losses and rarely offers access to all the elements involved at once, which complicates the quantitative analysis. However, on the other hand, the fine structure of the energy loss edges often allows to see interatomic bonds (energy loss near edge structure, ELNES) and the environment of the atoms (extended energy loss fine structure, EXELS) and to go beyond the simple elemental analysis. Both techniques use electronic probes of comparable diameters and should a priori offer the same spatial resolution. However, the EDXS detector has the disadvantage of collecting spurious X-ray photons created outside the analysis point by forward and back-scattered electrons, fast secondary electrons [34] and X-ray fluorescence, which adds a bias to the quantification estimate. The small electron collection angle of the EELS spectrometer allows information to be collected only from the forward scattered electrons in the immediate vicinity of the probe eliminating most, if not all, of the spurious signal and maintaining the spatial resolution close to the probe diameter.

In the present sample EDXS quantification requires an accurate deconvolution [35] of the overlapping characteristic lines Hf-M/Si-K and Hf-L/Cu-K and N-K/Ti-L. The amount of Ti was safely derived from its K line, while Ti-L3-M and Ti-L2-M are only3 and 10 eV above the N-K makes deconvolution for the nitrogen particularly difficult and unreliable. Line deconvolution was performed with the ESPRIT „serie fit” option [36] after energy recalibrations using Si-K and O-K or Cu-K and O-K lines from the substrate spectrum (Cu is always present due to scattered electrons to the supporting grid). Nevertheless, the absolute N concentration by EDXS remains subject to quite a large uncertainty, let’s say some ±10% at best due to systematic error in deconvolution. For that reason, it is better to compare the ratios of at. % concentrations between two elements (other than N) than at. % concentrations normalized to 100%.

Infrared (IR) absorption spectra were obtained in the range of 185–1400 nm (UV-Vis-NIR) from the TiN_x_ top surface and TiN_x_ bottom electrodes of the stacks using a Shimadzu UV-2600 spectrophotometer with the IRS-2600Plus integrating sphere (Shimadzu Corp.). The diffuse reflection spectra have been measured using integrating spheres with a spherical-shaped inner surface and inner wall made of barium sulphate light scattering material, having high reflectance. Recording was performed in reflection mode with an 8° radiation incidence and 2 nm spectral line width. Calibration was performed using the diffuse reflection from a cuvette made of the same material as the sphere (BaSO_4_). The band gap of the TiN_x_ top electrode was estimated by the Tauc method [37].

The sheet resistance of the top TiN_x_ film was measured at room temperature using the standard four-point probe with a Source Meter Keithley 2401 (Keithley Tektronix Ink). The temperature coefficient of resistance (TCR) was measured between 20 and 100 °C on a thermally stabilized stage (thermal chuck systems) using a thermocouple and a Multimeter Keithley 2010.

## 3. Results and Discussion

### 3.1. EDXS and EELS Measurements of O in TiN_x_ Top and Bottom Electrodes

**Figure 1:**
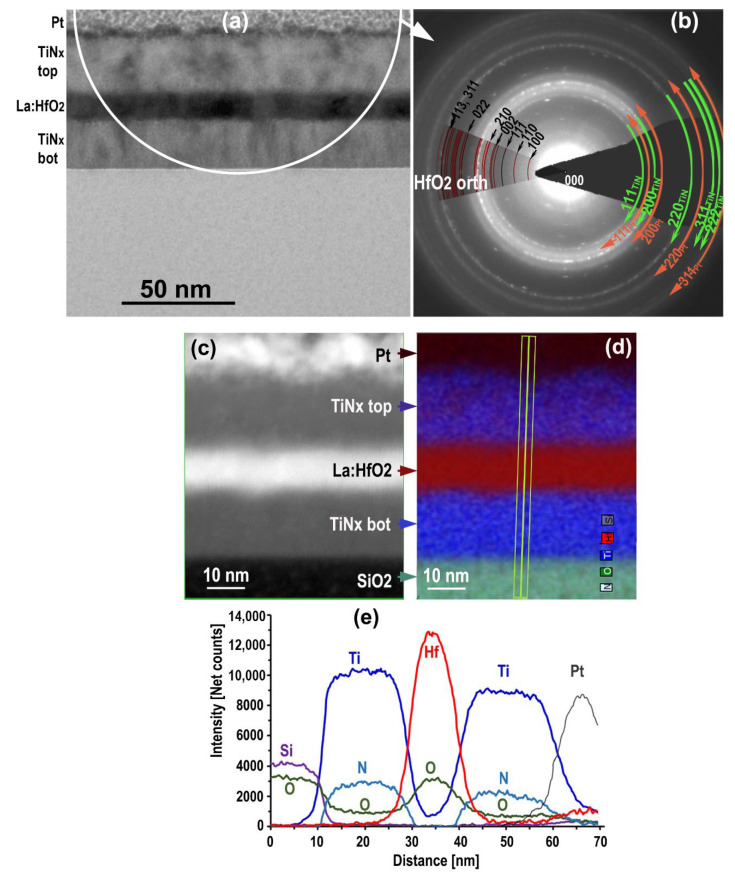
MIM structure on SiO_2_: Overall view of TEM bright field cross section (**a**), SAED pattern taken from the area highlighted contains labelled reflections from cubic TiN, Pt and nanocrystalline orthorhombic hafnium oxide (**b**); HAADF STEM image (**c**) and the corresponding EDXS element map with transverse line scan (**d**), Ti, N and O net counts profiles along the transverse line scan (**e**).

The bright field TEM image of Figure 1a shows that the upper and lower titanium nitride layers developed a columnar structure of some 5 nm width that led to a significant interface roughness. The roughness developed at the TiN_x_ propagates through the interface to the La:HfO_2_ layer and the advantage of the inherent low roughness of lanthanum doped hafnium oxide [38] is, at least severely, counterbalanced. The cubic FCC structure of TiNx is preserved, as shown by the SAED pattern in Figure 1b. Figure 1c,d show respectively a part view of the HAADF map in conditions of microanalysis and its corresponding EDXS map with the profile of the elements across the layer stock (Figure 1e). As mentioned above, the deconvolution quality of the overlapping N-K and Ti-L lines is below generally accepted limits and the confidence in the absolute nitrogen concentrations is low. Therefore, only ratios of net counts or at. % concentrations of oxygen to titanium are accurate in TiN_x_O_y_; stoichiometries from EDXS are approximate.

The compositions in the center of the TiN_x_ layers are constant within the experimental accuracy along an EDXS line scan at a mid-distance from the electrode interfaces. The relative O/Ti at. % average ratio amounts to 0.25 and 0.33 in the top and bottom layers, respectively. Taking into account nitrogen, approximate stoichiometric formulas would become TiN_0_._43_O_0_._25_ and TiN_0_._52_O_0_._33_. Monte-Carlo simulations with Casino 2.52 and McXRay 1.7 show that these values of oxygen content exceed by far the possible contribution of a FIB lamella surface oxidation before observation or a contribution due to scattered electrons out of the primary electron probe towards the oxygen-rich SiO_2_ substrate and the dielectric layer. This observation constitutes strong evidence of the presence of oxygen at the center part of the TiN layers.

Considering that the column has approximately the same extension around their axis, about 5 nm or less and that the sample is from 50 to 100 nm thick, EDXS is unable to tell if a new phase exists in addition to the superposition of the TiN_x_ and HfO_2_ layers, for instance, if a TiO_x_ or a ternary/quaternary Ti-N-Hf-O phase was formed. This ambiguity will be removed by the EELS study.

**Figure 2:**
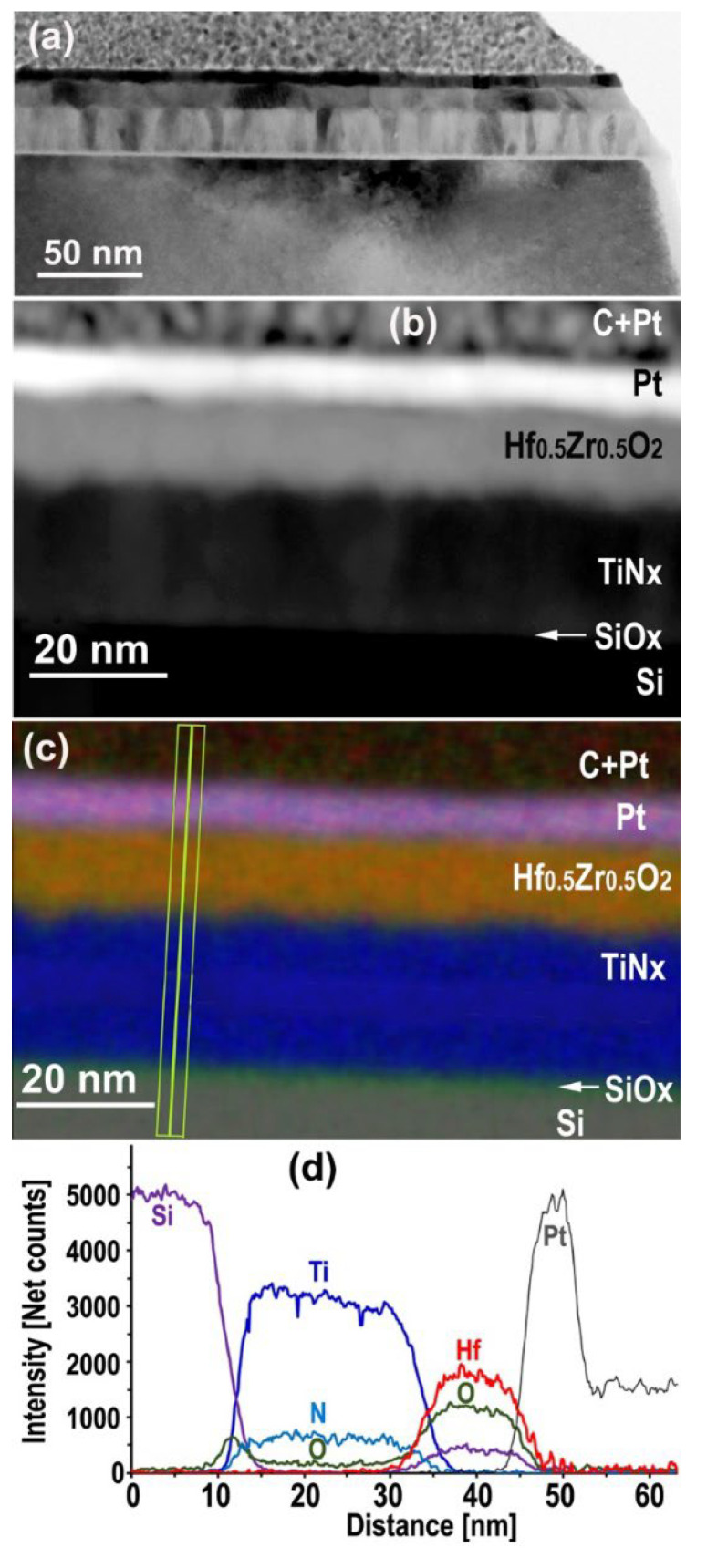
MIM structure on Si substrate with 1.5 nm native SiO_x_: Overall view of TEM bright field cross section (**a**), HAADF STEM image (**b**) and corresponding EDXS elements map with transverse line scans (**c**), Ti, N and O net counts transverse profiles along the line scan (**d**).

Among the available samples, a multilayer Si/1.5 nm Si-O native/20 nm TiN_x_/10 nm Hf_0_._5_Zr_0_._5_O_2_/4 nm Pt/Pt + C FIB cap was observed to compare the effect of a SiO_2_ substrate to a Si one with only 1.5 nm of native oxide on the oxygen content in the adjacent electrode (Figure 2a–c). The O-K/Ti-K ratio in the lower TiN_x_ drops to 0.17 (Figure 2d), which is about 51% of that observed on the SiO_2_ substrate (Figure 1e). Neglecting a possible contribution of oxygen from the ALD deposition process during the growth of the layer, a comparison between the two samples shows that half of the oxygen observed in the bottom TiNx layer with SiO_2_ comes from diffusion from the substrate and the other half from the HfO_2_ layer. Therefore, the HfO_2_ layer could also contribute in a symmetric way to 0.16 O/Ti in the top layer. Taking into account the oxygen incorporation during the ALD process, these values have to be reduced slightly.

According to Ahn et al. [39], oxygen left in the TiN_x_ layer on Si due to the plasma during growth amounts to about 0.04 O/Ti. In addition, to explain the difference remaining in the top layer of TiN_x_ between the observed 0.25 O/Ti concentration and the sum of the 0.16 O/Ti contribution of diffusion from HfO_2_ and plus the 0.04 O/Ti from ALD plasma contribution could be explained by oxygen diffusing along the grain boundaries [28,40] with an O peak at the surface (see section EELS) is added. Table 1 summarizes the measured concentrations (obs), the estimate from [39] and the inferred concentrations for balance (bal).

**Figure 3:**
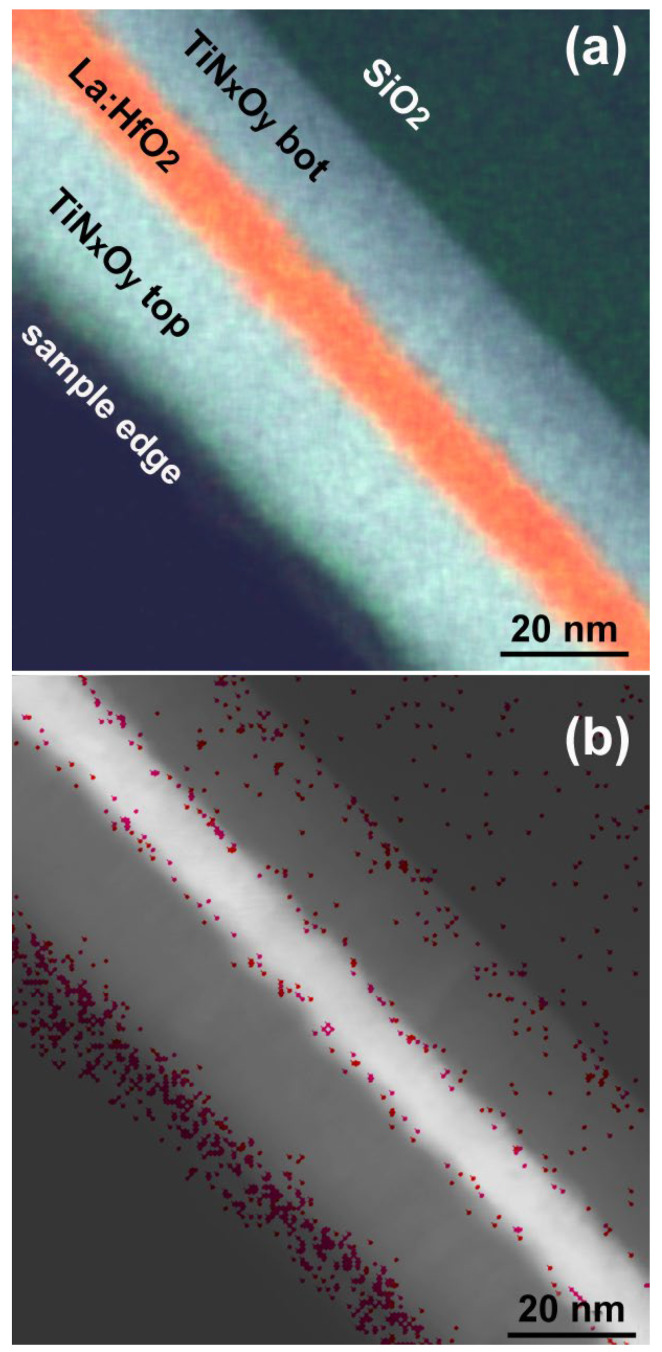
Image overlay of N, O, Si, Ti and Hf element map (**a**), HAADF STEM image with superimposed two-phase maps containing a maximum of Ti and O (**b**).

EDXS quantitative analysis at interfaces is hindered by the low number of X-ray counts available before unacceptable irradiation damage occurs as well as by the interface roughness that introduces blurring and prevents simple averaging along it. Principal component analysis (PCA)/histogram built-in ESPRIT software was used to establish correlations between X-ray counts from individual pixels in the EDXS map data cube. Adjusting the PCA sensitivity by limiting the number of components to 20 led to two phases simultaneously meeting the criteria Si < 2 at. % and N < 2 at. % and zero or negligible Hf. They appear mainly along interfaces (Figure 3) and their stoichiometries correspond to TiSi_0_._07_N_0_._06_O_2_ and TiSi_0_._04_N_0_._03_O_0_._88_. This strongly suggests that the interfaces can contain an interfacial phase close to TiO_2_. The EELS analysis below brings more details and confirms this interpretation.

As the energy resolution and sensitivity of EELS are better than EDXS for the analysis of light elements [41], we used core loss EELS to resolve the N-K, Ti-L and O-K overlapping lines in EDXS and to obtain more accurate concentrations of N together with Ti and O in TiN_x_O_y_ layers and their stoichiometry. In addition, the entrance aperture of the EELS spectrometer acts as a filter against electrons scattered out of the analysis point, which brings in spurious X-ray photons counted by the detector. As TiN degrades rapidly under the electron beam, a total of about 1000 spectra were collected at a reasonably low dose to obtain statistically significant data for background subtraction, deconvolution and concentration measurements.

**Figure 4:**
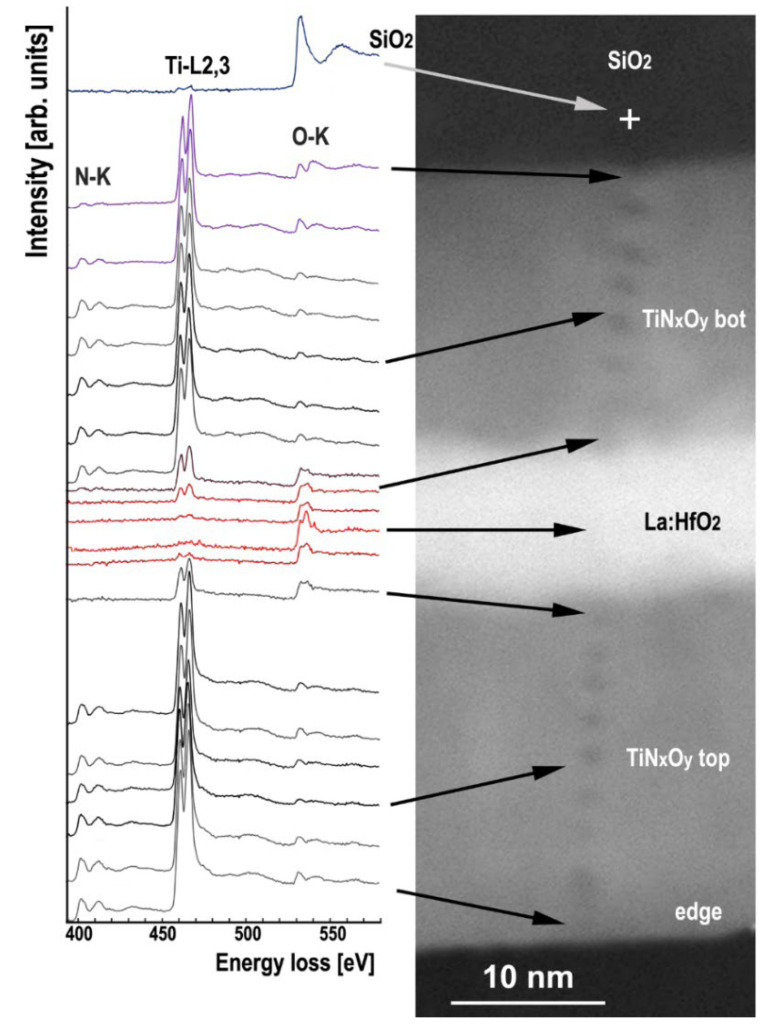
Set of EELS spectra in the range of 390–580 eV to show together the N-K (401 eV), Ti-L_2,3_ (456 eV) and O-K (532 eV) edges obtained across the SiO_2_—TiN_x_O_y_ bot—La:HfO_2_—TiN_x_O_y_ top interfaces with positions indicated by arrows in the corresponding HAADF STEM image; (spectra are shifted vertically for more readability).

Figure 4 (left) shows the 390–580 eV EELS spectra from which the N-K (401 eV), Ti-L_2,3_ (456 eV) and O-K (532 eV) edges were taken for element quantification using the power low background model.

In the HAADF-STEM image (Figure 4 right), dark spots correspond to irradiation damage at successive probe positions along a transverse path after 1 s acquisition of EELS spectra with a 1.5 nm probe. Their dark contrast in the TiN_x_ layers indicates a local reduction in sample thickness attributed to a moderate sputtering effect, not a carbonaceous contamination as often observed after exposure to an electron probe. After recalibrating the black level of the HAADF image for zero intensity over an out-of-sample area, quantitative contrast analysis in the top TiN_x_ layer indicates a sputtering depth of 7% and 13% of the pristine sample thickness, with mid-depth diameters of 1 and 1.3 nm for the lightest and darkest spots, respectively. The spots in the HfO_2_ layer are barely visible, confirming that TiN_x_ is much more sensitive to the irradiation effect than HfO_2_. This sputtering effect is unavoidable despite the effort made to minimize the risk of irradiation damage by defocusing the probe to spread the electron dose over the largest acceptable area while keeping it thin enough to maintain a spatial resolution of about 1 nm. In addition, a sufficient probe intensity is required to record spectra within about 1 s to avoid blurring by the sample drift.

**Figure 5:**
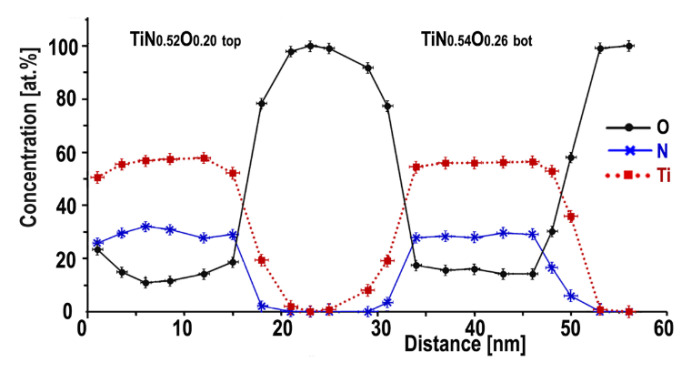
Concentrations in atomic % of N, Ti and O extracted from the line scan across the multilayered structure starting from the edge of TiN_x_O_y_ top. The tiny crosses are error bars.

The oxygen K-edge is observed in all spectra, confirming the presence of oxygen in the top and bottom titanium nitride electrodes. The concentrations of N, Ti and O determined by EELS were obtained using the Digital Micrograph 2.31 (Gatan, Pleasanton, CA, USA) quantification procedure and the concentration profiles are shown in Figure 5. Averaged over some 300 EELS spectra along line scans in the middle of the layer, the oxygen concentration is 14.2 at. % in the TiNxOy bottom and 11.4 at% in the TiNxOy top electrodes (standard deviations 0.1 and 0.5 at. %, respectively). The top surface of the TiN_x_O_y_ top is more oxidized and reaches about 25 at. %. This effect has already been reported [39,40,42].

The stoichiometry in both upper and lower TiN_x_O_y_ layers varies between 0.48 ≤ x ≤ 0.61 and 0.16 ≤ y ≤ 0.25. The average stoichiometric formulas at the center (far from interfaces) of the top and bottom electrodes can be written as TiN_0_._52±0_._04_O_0_._20±0_._05_ top and TiN_0_._54±0.04_O_0_._26±0.04_ bottom, respectively. Further, for sake of brevity, we continue to denote chemical formulas as TiN_x_O_y_ top or bottom.

**Figure 6:**
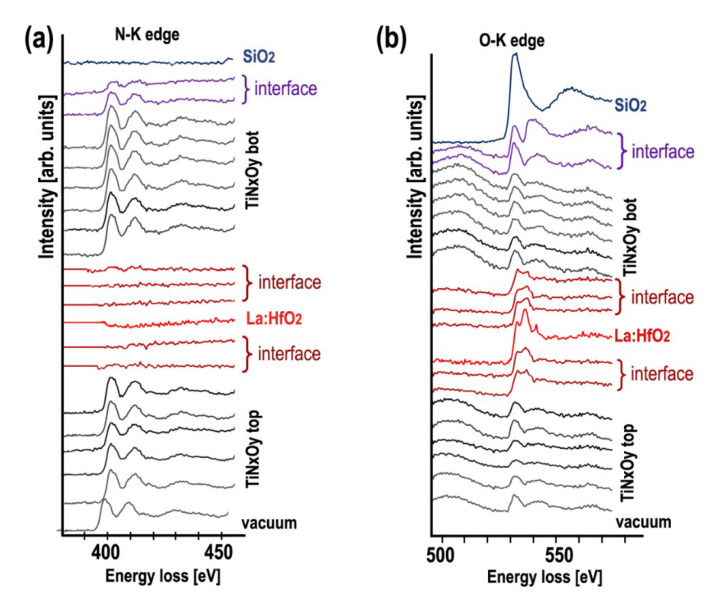
Evolution of ELNES through the SiO_2_—TiN_x_O_y_ bot—La:HfO_2_—TiN_x_O_y_ top interfaces: (**a**) oxygen K-edge and (**b**) nitrogen K-edge (spectra are shifted vertically for more readability).

**Figure 7:**
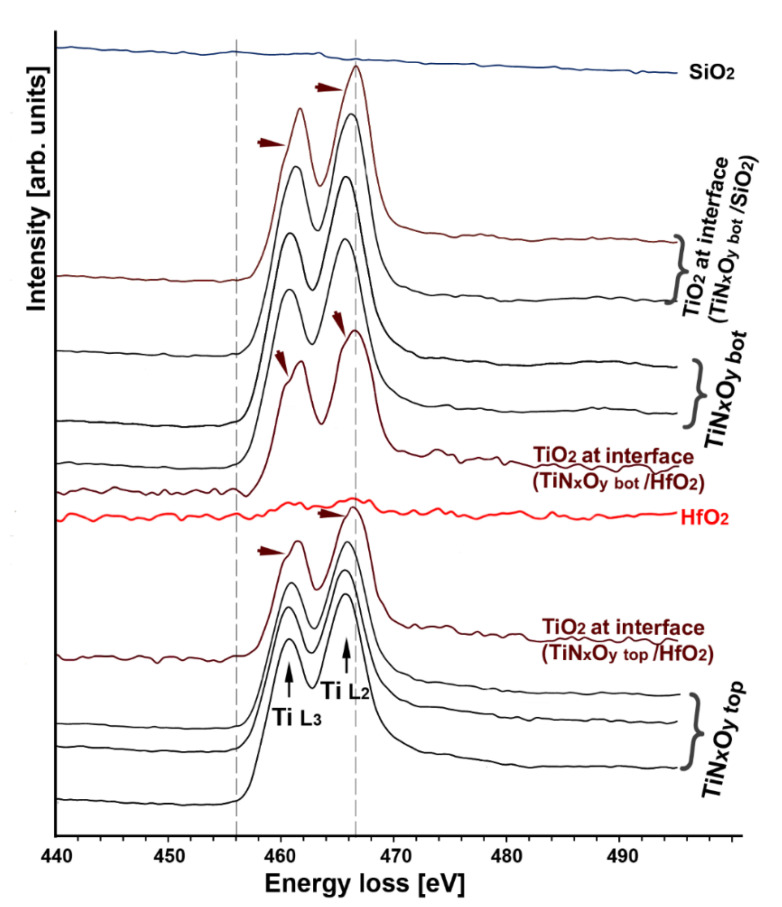
Shape evolution of the Ti-L_2,3_ edges across the SiO_2_—TiN_x_O_y_ bottom—La:HfO_2_—TiN_x_O_y_ top interfaces in the interfacial regions where a splitting in the Ti-L_2_ and Ti-L_3_ peaks (indicated by arrows) shows that TiO_2_ appears at the interface and chemical shift towards higher energy of the Ti L_2,3_ edges from titanium oxide (spectra are shifted vertically for more readability).

Figure 6 and Figure 7 show the evolution of the N-K, O-K and Ti-L_2,3_ core loss fine structures through the SiO_2_/TiN_x_O_y_ bot/La:HfO_2_/TiN_x_O_y_ top multilayer structure and its interfaces. The Ti-L_2,3_ core edges and energy onset in the center of the TiN_x_O_y_ layers in this work do not show significant differences compared to the spectra reported [43,44,45] on non-oxidized titanium nitride. The following particular features appear in spectra obtained near the interfaces: (i) at the interface with hafnium oxide, the presence of O-K and Ti-L2,3 edges while the N-K-edge has vanished is characteristic of the apparition of a TiO_2_ phase, (ii) the Ti-L_2,3_ edges in the spectra obtained at the TiN_x_ interface with La:HfO_2_ or SiO_2_ interfaces show a weak splitting and shift about 0.8 eV towards the higher energy region compared to the same edge in the central part of the TiN_x_O_y_ layers (Figure 7), (iii) all spectra close to interfaces are characteristic of a superposition of the two TiN_x_O_y_ and TiO_x_ phases.

The asymmetrical shape observed in spectra of the SiO2 interface with is due to a weak splitting of the Ti-L_2,3_ edges. The fine structure of the Ti-L_2,3_ edge is in good agreement with other publications for experimental and simulated from Ti oxide edges [44,45,46]. The experimental accuracy does not allow us to decide whether this asymmetry of Ti-L_2_._3_ edges is due to sub-nanometer crystalline or amorphous TiO_2_.

The structure of titanium oxynitride TiOxNy in thin films and bulk solids is considered to be a solid solution of FCC cubic TiN (a = 0.424 nm), TiN_0_._6_ (a = 0.420)4 and disordered FCC cubic TiO (a = 0.417 nm) [7,47,48,49]. The small difference between the crystal lattice parameters of oxynitride and pure TiN together with the limited electrode width on cross-section views makes it impossible to distinguish between them by selected area electron diffraction.

The incorporated oxygen ions predominantly occupy the positions of nitrogen vacancies near the grain boundaries, while vacancies remain inside the grains. This is evidenced by the non-stoichiometric oxynitride formula for both electrodes, where x and y vary from 0.48 ≤ x ≤ 0.61 to 0.16 ≤ y ≤ 0.25, i.e., Ti(N + O) 0.62–0.86.

The following several models that could explain the presence of oxygen in titanium nitride electrodes have been considered and discussed:

I. Post-growth transport: Results from numerous papers (for instance, [23,48]) show that exposure of TiN to air/oxygen flows leads to the formation of oxynitride and oxide at the surface and in its immediate vicinity. By the way, this effect was taken into consideration in the TEM sample preparation with a light Ar+ plasma cleaning (surface oxide removal [50]. In addition, oxygen may easily diffuse along the grain boundaries [28,40], bringing oxygen, i.e., deeper into the top electrode as far as the present work is concerned. However, such a mechanism will not completely explain the oxygen presence in the bottom electrode by diffusion through the dielectric. However, it could be understood if an RTA was performed at an elevated temperature that would add oxygen diffusion from the dielectric to both electrodes and possibly from the SiO_2_ substrate to the bottom electrode. According to experts in the microelectronics field, such high RTA temperatures would be incompatible with HfO_2_ in MIM structures integrated into devices, and therefore, the actual development of MIM with TiN and Hf progress without high-temperature RTA would not exist. Thus, the explanation for the oxygen presence must be sought elsewhere.

II. During growth: (i) Unexpected/uncontrolled sources may feed oxygen to the electrodes during MIM growth and possibly cooling. Oxygen could come from the gaseous reactant NH_3_ during the growth of TiN_x_ by PEALD or even from added carrier gas such as Ar, see, for instance [39,51]. In the latter one [51], an effort was made to clean the reactants from their impurities and concluded in a lowering of O in TiN down to 0.9%. According to Ahn et al. [39], such a source can bring no more than 4 at. %.

(ii): Transport from oxide layers towards electrodes: beyond the question of sufficient energy to break bonds in oxides to make oxygen available for thermal diffusion transport, another type of diffusion should be considered. For instance, in his study on the breakdown of MOS devices, Chen [52] reported the diffusion of oxygen from a high-k lanthanum oxide to the silicon electrode through the TiN gate electrode. Though the conditions are different from PEALD, this shows that TiN, in particular, if it is not stoichiometric, can be transparent to oxygen and allow oxygen transport at low temperatures under an electrical field. We propose here to consider a study on a possible plasma-enhanced diffusion to transport oxygen at intermediate temperatures (TiN_x_ ≈ 330 °C and HfO_2_ ≈ 225 °C) from the oxide layers to their TiN_x_ neighbors during the layer growth. It should be kept in mind that at the growth interface, hydrogen/nitrogen ions are present together with radicals. In addition, a sheath layer is present at the growing surface that increases the ions’ energy. For instance, Faraz et al. [53] showed that ions in oxygen plasma for TiO_2_ growth obtain energies of anaverage of 19 eV and a maximum of 27 eV, which are rather high enough to penetrate and interact with the underlying material at the nanometer scale.

Cottom et al. [29] in their theoretical study of the atomistic mechanism of oxygen migration to the interface between amorphous SiO_2_ and polycrystalline TiNx, they have shown that the arrival of oxygen atoms at the interface is accompanied by preferential oxidation of undercoordinated Ti sites at the interface, forming a Ti−O layer. Molecular dynamics and spin-polarized density functional theory calculations demonstrate the preferential incorporation of oxygen at defects within the TiN_x_ grain boundaries and their fast diffusion along them. N vacancies can affect the oxygen diffusion mechanism. It was proposed that the oxygen migrates and occupies the N vacancy site where it is then trapped due to the high energy barrier to be overcome to leave the vacancy site, i.e., 2.0 eV [29]. In the MIM structures studied, the numerous grain boundaries in the top/bottom electrodes significantly facilitate oxygen diffusion throughout the thickness of TiN_x_ layers, and the nitrogen-vacancy sites trap oxygen. All this leads to the formation of Ti(N + O) 0.62–0.86 oxynitride with the Ti oxide phase at the interfaces with hafnium and silicon oxides.

### 3.2. Measurements of Band Gap Energy, Resistivity, TCR

Depending on their composition and vacancy density, titanium nitrides and oxynitrides can behave as conductors or semiconductors, a status that plays an essential role as electrodes. The origin and details of this transition are discussed in several papers [49,54,55,56]. Therefore, part of this work aimed to determine the energy band gap of TiN_0_._52±0_._04_O_0_._20±0.05_/TiN_0_._54±0.04_O_0_._26±0.04_ with a high level of nitrogen vacancies using the low loss (valence) EELS (VEELS) and IR-vis-UV absorption spectra from the top electrode. In addition, it is assumed that the determination of the resistivity and the temperature coefficient of resistivity will show which status, conductor or semiconductor, TiN_x_O_y_ has. The use of two different types of methods to determine the bandgap values also constitutes a test of consistency.

**Figure 8:**
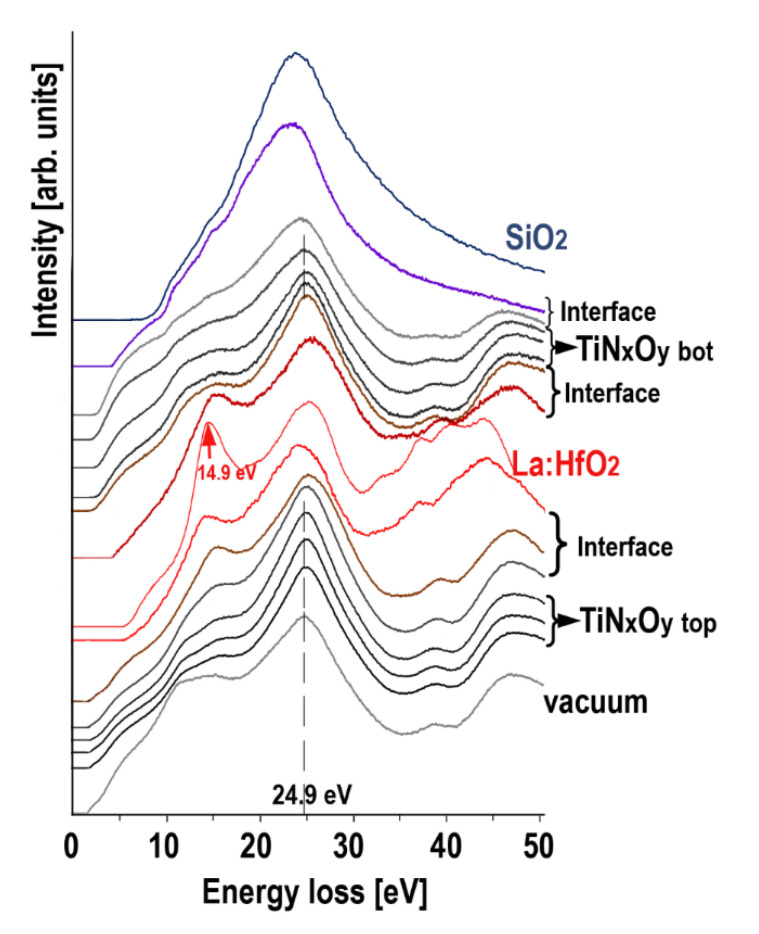
Energy loss functions obtained from the TiN_x_O_y_ top, La:HfO_2_, TiN_x_O_y_ bottom layers and SiO_2_ to estimate the energy band gaps; the 24.9 eV and 14.9 eV plasmon energies are given for TiN_x_O_y_ and of for La:HfO_2_, respectively.

The band gap width (the energy interval without intensity) was derived from the energy loss functions (ELF) presented in Figure 8 for single scattering distribution after subtracting the zero-loss peak and removing plural scattering.

The first observation that can be deduced from Figure 8, demonstrating the modification of the ELF through the multilayer structure, is that the peak of plasmon energy for TiN_x_O_y_ top and TiN_x_O_y_ bottom is the same and remains at 24.9 eV. It means that the valence electron density with some random noise also remains unchanged. The full width at half maximum (FWHM) of the plasmon for TiN_x_O_y_ bottom and TiN_x_O_y_ top is identical, and the FWHM expands only on direct contact with the hafnium oxide. Since the FWHM is related to the lifetime of the plasma oscillations [57], the damping coefficient is relatively isotropic for both titanium oxynitride layers despite the small difference in oxygen content. Figure 8 also shows the low loss spectrum attributed to pure La:HfO_2_, which exhibits the following two characteristic features: (i) a bulk plasmon peak at 14.9 nm and (ii) single-particle interband transitions that contribute to the oscillator strength at about 24.9–25.0 eV, which has already been observed [33,58].

**Figure 9:**
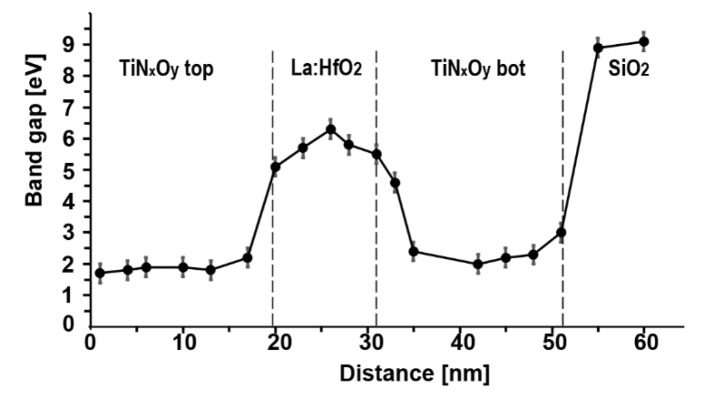
Variation in the band gap energy across the multilayer structure.

The profile of the band gap values through the TiN_x_O_y_ top/La:HfO_2_/TiN_x_O_y_ bot/SiO_2_ MIM is shown in Figure 9. The 1.7 eV lowest band gap is observed at a distance of 1–1.5 nm from the surface of TiN_0_._52_O_0_._20_ top. It increases to 2.0 eV at the center of this electrode. In the interfacial regions, it quickly raises up to 6.0–6.1 eV in the center of the La:HfO_2_ layer. Beyond the interfacial region with the bottom electrode, the band gap at the center of TiN_0_._54_O_0_._26_ bottom is 2.2–2.5 eV before an abrupt jump to 8.8–8.9 eV in the SiO_2_ layer.

**Figure 10:**
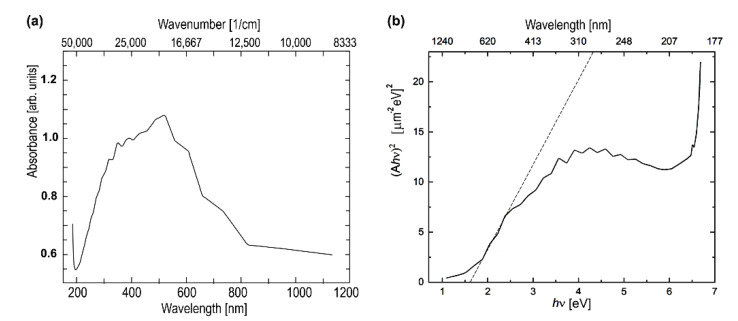
Absorption spectrum obtained from the top TiN_x_O_y_ electrode (**a**), determination of the band gap energy *E_g_* from IR-vis-UV absorption spectra (**b**).

The IR-vis-UV absorption spectrum used to measure the band gap width on the TiN_x_O_y_ top electrode is shown in Figure 10a. Due to the stacking of several thin films of different thicknesses in TiN_x_O_y_ top/La:HfO_2_/TiN_x_O_y_ bot/SiO_2_, an interference pattern was obtained, which was eliminated by averaging the spectra, knowing the thickness of all films, and assuming that the interference is associated with a TiN_x_O_y_ film in the wavelength range of 700–1200 nm and with La:HfO2 in the range of 200–600 nm.

An estimate of the band gap width in the top layer was obtained from the absorption spectra in accordance with the equation ${\left(\alpha \cdot h\nu \right)}^{\gamma }=A\cdot \left(h\nu -E_{g}\right)$ derived in [37] where α is the dependence of the absorption coefficient from the energy of incident radiation, *h* is Planck’s constant, *ν* is the photon frequency, *E_g_* is band gap energy, *A* is a coefficient depending on the effective mass of carriers and *γ* is a coefficient depending on the material. For our estimation, *γ* =2 was taken into consideration that TiN [59] and Ti oxynitride [60] are direct-band-gap semiconductors. The band gap width of about 1.6 ± 0.1 eV for the top electrode was derived by extrapolating the linear section of the dependence (*α·hν*)^2^ = f (*hν*) from *hν* to the zero-absorption coefficient (Figure 10b). This value of the band gap energy agrees well with the results obtained from VEELS for the TiN_x_O_y_ top layer (Figure 9).

These results are in agreement with Xie et al. [61], who have reported band gaps of from 1.68 to 3.23 eV for a TiO_2_-TiN composite film. In addition, our results for the band gap ranging between 2.2 and 2.5 eV for the bottom and 1.7 and 2.2 eV for the top TiN_x_O_y_ electrodes, respectively, confirm the values of 2.42–2.62 eV for Ti_2_N_2_O estimated by Aoki et al. [60] using the first principles many-body perturbation theory based on the GW approximation.

Our four-point probe measurements of the TiN_x_O_y_ top electrode gave a sheet resistance R = 104 Ω/sq, which corresponds to a resistivity ρ = 208 µOm⋅cm. An electrical resistance of 250 ohms/cm was achieved in a similar PEALD titanium nitride electrode obtained at 320 °C [62]. The slight difference in the resistivity measured in our work can be explained by the higher growth temperature of the layers, which contributes to the more efficient removal of chlorine impurities and a slightly larger grain size. Comparison with other known results shows that the PEALD top electrode has a higher resistivity than standard polycrystalline titanium nitride bulk samples of 30 µOhm⋅cm [63], 70 µOhm⋅cm [64] or 90 ± 10 µOhm⋅cm [65], but lower than Ti oxynitride films of 300 µOhm⋅cm [66] or 700 µOhm⋅cm [67] or slightly oxidized stoichiometric TiN films of 290 µOhm⋅cm [68]. These differences in resistivity may be due to changes in the columnar structure and composition of TiNx (not only N content but also O, H, C and Cl impurities).

The temperature coefficient of resistance (TCR) measured in the temperature range of 20–100 °C is negative (−31.4·10^−6^) 1/K and indicates a semiconductor nature of the electrodes.

The data presented here show that TiN_x_O_y_ electrodes can fulfill their electrical functions. Replacing them with ruthenium electrodes [69] has improved the endurance of the stacks, but so far has not led to a significant increase in reliability and is accompanied by large fatigue, which is usually associated with the structural characteristics of materials. It is obvious that further research on these materials is still needed, in particular, to identify even more precisely the critical parameters and to improve the technique to ensure their obtention in production conditions.

## 4. Conclusions

To summarize, the suspected presence of a significant amount of oxygen has been confirmed in all layers of ALD SiO_2_/TiN_x_/La:HfO_2_/TiN_x_ MIM stacks and a sub-nanometer thick TiO_2_ layer has appeared at the TiN_x_/oxide interfaces. From the comparison of the oxygen content in the bottom TiN_x_ electrode grown on SiO_2_ or Si/1.5 nm native oxide, the contribution to the concentration observed in TiN_x_ from the oxygen impurity of the NH_3_ plasma itself appears to be only minor. We suggest that an oxygen diffusion stimulated by the plasma ions and radicals may already occur during PEALD deposition at fairly low temperatures from the existing oxides (substrate, then dielectric). Oxygen compositions measured by EELS spectroscopy at the core of the top and bottom electrodes are about 14.2 at% and 11.4 at%, respectively, which correspond to TiN_0.52±0_._04_O_0_._20±0_._05_ top and TiN_0_._54±0_._04_O_0_._26±0_._04_ bottom stoichiometries. The at% oxygen concentration normalized to titanium O/Ti determined by EDXS spectrometry are 0.25 and 0.33 for these electrodes. Using an alternate stack with a Si-native oxide substrate instead of SiO_2_, the bottom O/Ti drops to 0.17. This difference is attributed to the absence of oxygen diffusion from the Si substrate to the electrode. It shows that oxygen diffusion from the HfO_2_ layer is similar to that from the SiO_2_ substrate. In addition, the value of 0.04 O/Ti for oxygen incorporation during ALD growth would be low in comparison with the 0.33 total for the bottom electrode and still leave 0.08 O/Ti for the top electrode. The splitting of the Ti-L_2,3_ edges together with their 0.8 eV chemical shift clearly indicates the presence of TiO_2_ at the interfaces with La: HfO_2_ and SiO_2_.

Measurements of band gaps in TiN_x_O_y_ with VEELS show that their values are in the range of 2.2–2.5 eV for the bottom TiN_x_O_y_ and 1.7–2.2 eV for the top TiN_x_O_y_, which is in fair agreement with the value of 1.6 eV obtained in the top electrode using optical absorption spectra. Measurements on the top TiN_x_O_y_ electrode of the four-point probe sheet resistance, the resistivity and temperature coefficient of resistance from 20 to 100 °C show values characteristic of semiconductors.

## Figures and Tables

**Table 1:** Relative atomic % content O/Ti in TiN_x_.

Substrate	SiO_2_	Si + O-native	SiO_2_
Layer	Bottom	Bottom	Top
O/Ti total	0.33 (obs)	0.17 (obs)	0.25 (obs)
O/Ti—Diffusion from substrate	0.16 (bal)	---	---
O/Ti ALD contribution	0.04 [39]	0.04 [39]	0.04 [39]
O/Ti—Diffusion from HfO_2_	0.13 (bal)	0.13 (bal)	0.13 (bal)
O/Ti—Diffusion from surface	---	---	0.08 (bal)

## Data Availability

Data are available at reasonable request from the authors.
